# *Peumus boldus* Used in the Synthesis of ZnO Semiconductor Nanoparticles and Their Evaluation in Organic Contaminants

**DOI:** 10.3390/ma16124344

**Published:** 2023-06-13

**Authors:** Caree Abigail García Maro, Horacio Edgardo Garrafa Gálvez, Osvaldo de Jesús Nava Olivas, Mizael Luque Morales, Diana Vargas Hernández, Hugo Galindo Flores, Víctor Manuel Orozco Carmona, Manuel de Jesús Chinchillas Chinchillas

**Affiliations:** 1 Facultad de Ingeniería Mochis, Universidad Autónoma de Sinaloa, Fuente de Poseidón y Prol. Ángel Flores S/N, Los Mochis C.P. 81223, Mexico; caree.garcia@uas.edu.mx (C.A.G.M.); horacio.garrafa.fim@uas.edu.mx (H.E.G.G.); 2 Centro de Nanociencias y Nanotecnología, UNAM, Ensenada 22860, Mexico; navao@uabc.edu.mx; 3 Facultad de Ingeniería, Arquitectura y Diseño, Universidad Autónoma de Baja California, Ensenada C.P. 22860, Mexico; mizael.luque@uabc.edu.mx; 4 Instituto Tecnológico Nacional, Campus Guasave, Guasave C.P. 81149, Mexico; 5 Departamento de Investigación en Polímeros y Materiales, CONACYT-Universidad de Sonora, Blvd. Luis Encinas Johnson y Rosales S/N, Hermosillo 83000, Mexico; dvargashe@conacyt.mx; 6 Departamento de Ingeniería y Tecnología, Universidad Autónoma de Occidente (UAdeO), Guasave 81048, Mexico; hugo.galindo@uadeo.mx; 7 Departamento de Metalurgia e Integridad Estructural, Centro de Investigación en Materiales Avanzados (CIMAV), Av. Miguel de Cervantes Saavedra 120, Complejo Industrial Chihuahua, Chihuahua 31136, Mexico

**Keywords:** biosynthesis, *Peumus boldus*, organic pollutants, photocatalysis

## Abstract

The high demand for nanomaterials in the field of industry and science has forced researchers to develop new synthesis methods that are more efficient, economical, and environmentally friendly. At present, the application of green synthesis has taken a great advantage over conventional synthesis methods because it helps with the control of the characteristics and properties of the resulting nanomaterials. In this research, ZnO nanoparticles (NPs) were synthesized by biosynthesis using dried boldo (*Peumus boldus*) leaves. The resulting biosynthesized NPs had a high purity, quasi-spherical shape with average sizes ranging from 15 to 30 nm and a band gap of ~2.8–3.1 eV. These NPs were used in the photocatalytic activity of three organic dyes. The results showed degradation of 100% methylene blue (MB) in 180 min, 92% methyl orange (MO) in 180 min, and 100% Rhodamine B (RhB) in 30 min of exposure. These results show that the *Peumus boldus* leaf extract is effective in the biosynthesis of ZnO NPs with good photocatalytic properties.

## 1. Introduction

Currently, the cost of generating or synthesizing semiconductor nanomaterials is high for most industries, which is why, lately, researchers are looking for alternatives to change or modify current procedures and use more accessible and lower-cost materials. That is why the use of materials from nature (in some cases waste) has gained great importance. Some plants, roots, and leaves have been widely used in medicine because these materials have compounds that can help with hypertension, diabetes, kidney problems, and cancer-related problems, among others. [1,2,3]. On the other hand, they have also been used in the construction industry since it has been reported that the use of materials such as nopal mucilage [4], marble, walnut shells [5], and palm leaves [6], among others, manages to increase the mechanical properties of materials, such as resistance to compression, flexibility, durability, etc. In the synthesis of nanomaterials, such as nanoparticles (NPs), the use of natural materials has attracted attention in the scientific community because it is possible to substitute some chemical reagents that are harmful and dangerous to people’s health and the environment [7]. The process of exchanging hazardous reagents for natural materials, minimizing energy, reducing waste, and reusing solvents and materials is known as green chemistry [8]. This new process has been reported to bring great advantages, being cheaper, simpler, faster, and less dangerous for the environment. Within green chemistry for the synthesis of NPs, there is a great diversity of elements that can be used, such as bacteria, whole plants, roots, branches, and leaves of trees, fruits, and/or vegetables [9]. All these elements present a set of organic molecules such as flavonoids, polyphenols, vitamins, enzymes, carbohydrates, etc., which are necessary to be able to carry out the process of the formation of NPs [10,11,12]. Nabi et al. synthesized TiO_2_ NPs by green synthesis using cinnamon tea extract (*Cinnamomum*), achieving nanomaterials with sizes of 70–150 nm [13]. Elsewhere, P.A. Luque et al. used lemon peel extract (*Citrus aurantifolia*) to biosynthesize SnO_2_ NPs with sizes of 5–12 nm [14]. An organic source that has been little reported despite its great benefits is the dried leaf of the boldo plant (*Peumus boldus*).

*Peumus boldus* is a native tree of Chile and its use began from the first natives to the present day. The leaves of this tree have been used to reduce earaches, headaches, rheumatism, nasal congestion, and digestive and biliary disorders [15]. Its use has also been associated as an antibacterial, antifungal, insecticide, anticancer, and antioxidant [16]. There are various applications for the leaves of *Peumus boldus* as the leaves of this tree contain various useful molecules, such as alkaloids (boldine, N-methylcoclaurine, isoboldine, (−)−pronuciferine, coclaurine, etc.), flavonoids (isorramnetin 3-O-glucoside-7-O-rhamnoside, ramnetin 3-O-arabinosidse 3’-O-rhamnopyranoside, etc.), phenolic compounds, and essential oils [17]. 

Within the wide range of NPs, those of semiconductor oxides are being widely used in various applications because they contain good mechanical, optical, electrical, catalytic, and magnetic properties [18]. Through green synthesis, the biosynthesis of CeO_2_ (Cerium oxide NPs), AgO_2_ (Silver oxide NPs), TiO_2_ (Titanium oxide NPs), WO_3_ (Wolfamium oxide NPs), Co_3_O_4_ (Cobalt oxide), SnO_2_ (Tin oxide), and ZnO (Zinc oxide) can be used for biological applications [19], antibacterial activity [20], antimicrobial activity [21], antifungal activity [22], battery improvement [23], and photocatalysis [24,25], among others. ZnO NPs are widely used in the scientific world because they have good properties, such as high transparency, high electron mobility, high luminescence, are biocompatible with other materials, and are ecological, in addition to having an approximate band gap of 3.3 eV, which can be very useful in photocatalysis [26]. To date, some organic sources have already been reported in the green synthesis of ZnO NPs, such as the one reported by Moghaddas et al. in 2020 who used Quince Seed Mucilage (QSM) to obtain ZnO NPs, which were used in the photocatalytic degradation of MB [27]. In addition, Luque-Morales et al., 2021 reported the successful biosynthesis of ZnO using Anaheim chili extract (*Capsicum annuum* var. *Anaheim*), thereby showing that the NPs obtained present photocatalytic activity for the degradation of different organic pollutants [28].

Photocatalysis is an effective method to achieve the removal of pollutants present in wastewater using solar energy or ultraviolet energy. In this procedure, the electrons in the valence band are agitated, jumping to the conduction band, generating an electron-hole pair. These chemical species interact with the pollutant molecules, causing their degradation [29,30]. Procedures such as this are currently necessary due to the high pollution generated in the industry, with the waste of organic dyes in aquifers, which causes problems for aquatic life (flora and fauna), and consequently for human health [31]. There is a wide variety of organic dyes; about 800,000 tons of dyes are produced each year, with the textile industry using 10 to 15% of them [32]. Within these dyes, MB, MO, and RhB are the most used in many industries and have a complex chemical structure, which makes their natural degradation process difficult [33]. Therefore, this method of photocatalysis helps to completely degrade or decrease the concentration of these dyes in the water, up to the permissible limits set by some international standards. 

In this study, the results of the green synthesis of ZnO NPs using dried boldo (*Peumus boldus*) leaves as a reducing and stabilizing agent are presented. This work follows up on previous studies reported by the authors using different organic sources. The obtained NPs were characterized by ATR-IR, XRD, XPS, TGA, BET, XRD, SEM, TEM, and Uv-Vis (band gap), and used to degrade three different organic pollutants (MB, MO, and RhB) under ultraviolet radiation.

## 2. Materials and Methods

### 2.1. Materials

The materials used in the biosynthesis were dry *Peumus boldus* leaves locally purchased in Ensenada, Baja California. Deionized water, with a pH of 6.8 (±0.2), from Sumilab, B.C. The Zinc nitrate [Zn(NO_3_) 2·6H_2_O], at a 98% purity, and the organic dyes were purchased commercially (Sigma Aldrich, St. Louis, MO, USA). Methylene Blue (MB) with a molecular weight of 373.9 g/mol and a purity of 90%, Methyl Orange (MO) with a molecular weight of 327.34 g/mol and a purity of 99%, and Rhodamine B (RhB) with a molecular weight of 479.01 g/mol and a purity of 95%. 

### 2.2. Extract Preparation 

Three different Extracts were prepared using different amounts of dried Boldo leaves: 1, 2, and 4 %, (0.5, 1, and 2 g, respectively), relating to the amount of water (weight/volume ratio). The steps to acquire the extract are: (1) Washing, drying, and crushing the *Peumus boldus* leaves and mixing them with deionized water (50 mL). (2) The blend is then stirred for 2 h at room temperature. (3) The blend is then placed inside a sous vide for 1 h at 60 °C. (4) The blend is then filtered through Whatman #4 paper.

### 2.3. Nanoparticles Synthesis 

Once the extracts were obtained, biosynthesis is as follows: (1) 2 g of zinc nitrate is added to each extract, then stirred for 1 h in the dark. (2) The mixes are placed in a sous vide for 12 h at 60 °C, sometimes more, depending on the consistency of the resulting material. (3) The different mixes are placed in porcelain dishes and calcined for 1 h at 400 °C. (4) Using an agate mortar, the calcine samples are pulverized for ease of use. The samples were identified as follows: 1% PB-ZnO, 2% PB-ZnO, and 4% PB-ZnO.

For the preparation of the extracts and the synthesis of the NPs, the methodology reported by the authors was followed, considering some variations [28]. 

### 2.4. Characterization

The ZnO NPs were characterized via ATR-IR, using equipment from Perkin Elmer (0.5 cm^−1^), to study the bonds in the functional groups present within the NPs. Thermal behavior was evaluated by TGA/DSC with a TA Instrument-SDT Q600 unit (temperature ramp from room temperature to 800 °C rising at 10 °C/min). The specific surface area and pore size were evaluated using the Brunauer–Emmett–Teller (BET) method with a TriStar II 3020 equipment (N_2_ adsorption at 77 K). The morphology of the nanomaterials was also evaluated by SEM-EDS with a JEOL-JSM-6310LV unit (working distance of 10 mm). The morphology and particle sizes were obtained through TEM using JEOL-JEM-2100 equipment (125 kV acceleration). On the other hand, the crystalline structure was evaluated using a Bruker-D2 Phase XRD (at 30 kV, 10 mA, and a range from 10 to 80 2θ). The XPS analysis was made with a Thermofisher Escalab 250Xi (Using monochromatic Al K Alpha radiation, and a step energy of 20 eV). Additionally, the band gap value was calculated using the TAUC model by means of the UV-Vis spectrum obtained from Perkin Elmer-Lambda 365 equipment (wavelength from 190 to 800 nm, and resolution speed of 600 nm/min).

### 2.5. Photocatalytic Activity

The photocatalytic study was carried out under ultraviolet radiation using Polaris UV-1C brand reactors. The first step to carry out the photocatalytic study was the preparation of MB, MO, and RhB solutions. In 50 mL of water, 15 ppm of the organic dyes were added to different reactors, then 50 mg of NPs were added to each solution and stirred for half an hour in the dark. Subsequently, the UV lamps were turned on and the process began. A total of 2 mL of each solution was withdrawn for evaluation every 10 min during exposure for the first hour, and every 20 min for the next two hours. The concentrations of the solutions were evaluated by UV-Vis spectroscopy.

## 3. Results and Discussion

### 3.1. ATR-IR

**Figure 1:**
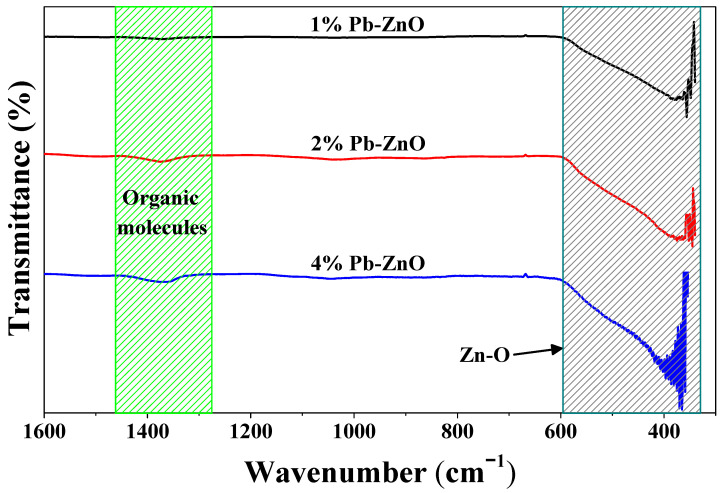
ATR-IR of the ZnO NPs biosynthesized with *Peumus boldus*.

The vibration between the bonds of the ZnO NPs biosynthesized with *Peumus boldus* is observed in Figure 1. At first glance, the wide band found from 600 to 370 cm^−1^ in the three study samples stands out. This broad band corresponds to the vibration of the Zn-O bonds present in the nanoparticles [34]. In addition, other vibrations are identified at 1385 and 1050 cm^−1^, approximately. These vibrations are assigned to the organic molecules within the *Peumus boldus* extract [35,36]. As the percentage of extract used in the biosynthesis of the NPs increases, the bands assigned to the organic molecules increase in intensity, which indicates that some molecules remain attached to the ZnO NPs after the biosynthesis process. This could help in the photocatalytic process.

### 3.2. TGA

**Figure 2:**
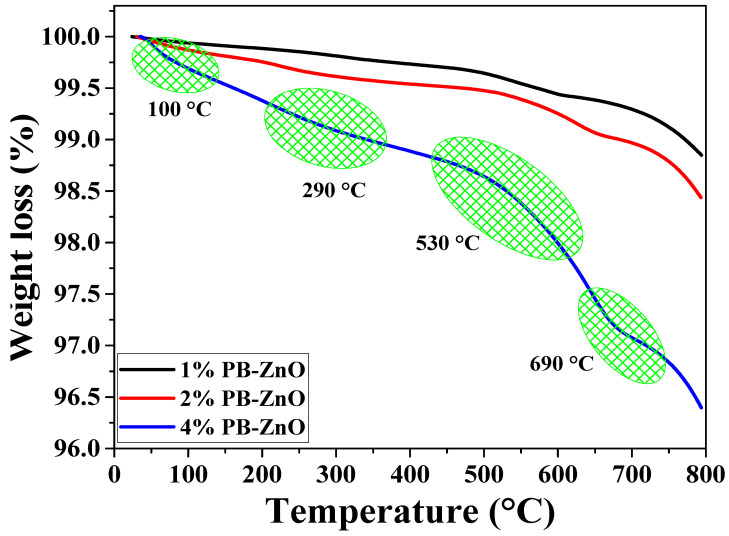
TGA analysis of ZnO NPs biosynthesized with *Peumus boldus*.

The thermal behavior of the NPs up to 800 °C is observed in Figure 2. The behavior of the 1%PB-ZnO and 2% PB-ZnO samples is very similar, with a weight loss of 1.2 and 1.6%, respectively. On the other hand, the 4% PB-ZnO sample presented several weight losses throughout the thermogravimetric analysis due to the large number of organic molecules present in the nanomaterial. The TGA profile of this sample shows a total weight loss of 3.6%. The most significant losses are found at approximately 100, 290, 530, and 690 °C. The first loss is recognized as the evaporation of water physically bound to the molecules present in the ZnO NPs, while the other weight losses are attributed to the removal of organic material and the formation of ZnO nanoparticles [37,38].

### 3.3. SEM-EDS

**Figure 3:**
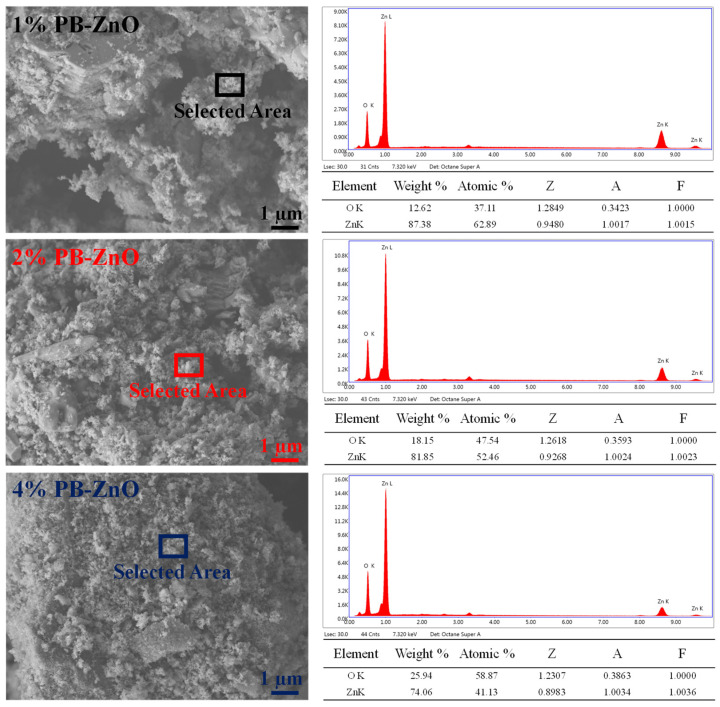
SEM/EDS of ZnO NPs biosynthesized with *Peumus boldus*.

Figure 3 shows the morphological study of the ZnO NPs that were biosynthesized with *Peumus boldus*. The NPs with 1% extract have larger granules or crystalline agglomerations than the other samples in the study. It is also notable that, as the amount of extract used during the biosynthesis increases, the crystals and agglomerations decrease, with the sample with 4% (4%PB-ZnO) showing the least agglomeration and the most separated NPs. This shows that the organic molecules present in the NPs help in the process to control the size of the NPs [39]. Taking advantage of the technology, EDS analysis was carried out, showing that the atomic percentage of oxygen increases proportionally to the percentage of the extract in biosynthesis, which could indicate that there is a greater amount of this element due to the extract used [40].

### 3.4. TEM

**Figure 4:**
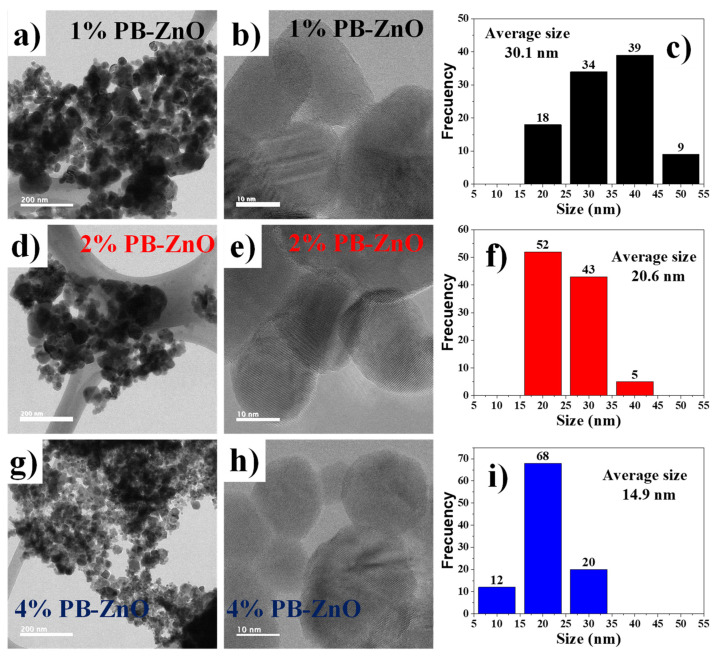
TEM of biosynthesized ZnO NPs with *Peumus boldus*.

To observe in detail the shape of the ZnO NPs biosynthesized with *Peumus boldus*, a TEM study was carried out, which is shown in Figure 4. Figure 4a–c correspond to the 1% PB-ZnO sample, in which it is possible to observe NPs that have an elongated, quasispherical morphology, with very little agglomeration. The NPs showed an average diameter of 30.1 nm, with sizes between 20 and 50 nm. Other studies have reported similar results [41]. Next, Figure 4d–f present the information of the NPs biosynthesized with 2% *Peumus boldus* extract, where it is possible to observe NPs with a more spherical shape and little agglomeration. The average size of these NPs was 20.6 nm. Finally, Figure 4g–i show the biosynthesized NPs with 4% extract. It is notable that the NPs are smaller in size than the other study samples, having an average diameter of 14.9 nm. The morphology is quasispherical, and a more notable separation is observed between each particle [42]. As presented in the study, there is an inversely proportional decrease in the size and a greater separation between the NPs as the percentage of extract used in the biosynthesis increased. This is attributed to a greater quantity of organic molecules involved in the growth process of the NPs, which causes greater nucleation during the metal salt reduction process, resulting in a greater number of smaller particles. In addition, other studies have reported that organic molecules could function as a barrier to prevent agglomeration [43].

### 3.5. BET

**Figure 5:**
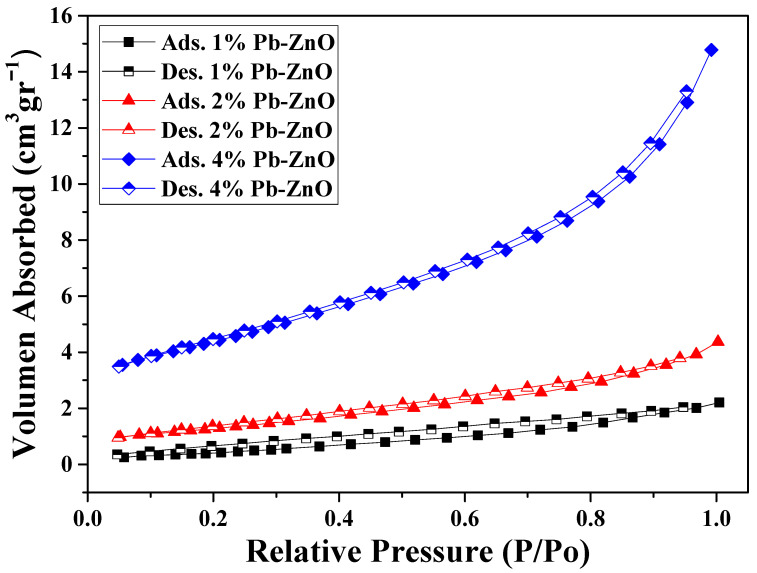
Nitrogen adsorption/desorption isotherms in the BET study.

The surface analysis of the biosynthesized ZnO NPs (shown in Table 1) presents the results of pore size and volume, as well as the surface area of the NPs. The surface area of the samples with 1, 2, and 4% of extract used showed a value of 1.81, 4.64, and 8.35 m^2^/g, respectively. As previously mentioned in the TEM study, as the percentage of extract used for the biosynthesis of ZnO NPs increases, the size of the NPs decreases. This occurs because it contains a greater amount of organic molecules which react with the metal salt and decrease its size [44]. Other studies have reported that the surface area of commercial ZnO NPs is around 3.23 m^2^/g, which indicates that it is possible to obtain NPs with a 2.5 times higher surface area via green synthesis with *Peumus boldus* [45]. Moreover, the pore volume could be related to the surface area; the larger the detected surface, the more pores are detected in the process. Finally, Figure 5 shows the N_2_ absorption/desorption isotherm of the study. The hysteresis loops that appear from 0.6 P/P0 indicate the presence of mesopores, mainly in the 4%PB-ZnO sample, indicating that the hysteresis shape is related to the texture of the NPs (pore size distribution, geometry, and connectivity) [46].

### 3.6. XRD

**Figure 6:**
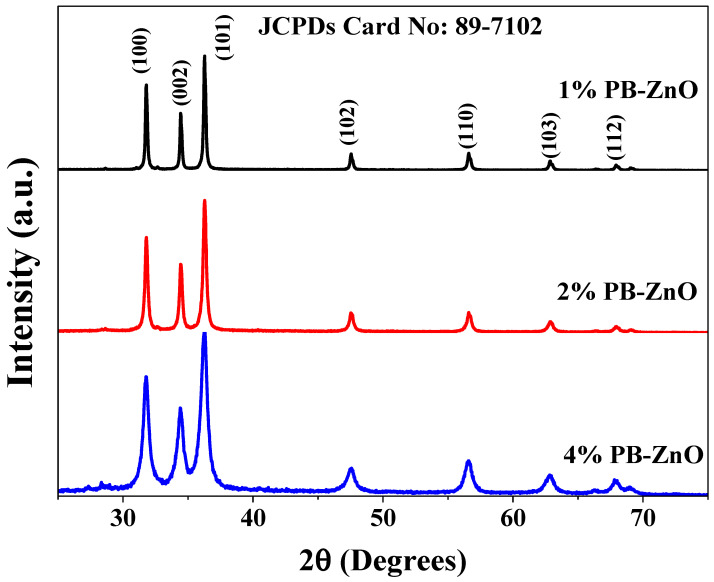
XRD spectra of the ZnO NPs biosynthesized with *Peumus boldus*.

The crystallographic structure of the ZnO NPs biosynthesized with *Peumus boldus* is shown in Figure 6. The XRD analysis shows seven peaks at 31.79, 34.43, 36.28, 47.5, 56.60, 62.87, and 67.93 2*θ* corresponding to the (100), (002), (101), (102), (110), (103), and (112) planes, respectively. This crystal arrangement denotes that the ZnO NPs have a hexagonal Wurtzite crystal structure, matching the file of the Joint Committee on Powder Diffraction Standards (JCPDS:89-7102) [47]. Furthermore, the crystallite size was calculated using the Scherrer equation (Equation (1)).

(1) $$D=\frac{K\lambda }{BCos\theta }$$

where *D* is the physical size of the crystallite at the diffraction peak, *B* is the peak bandwidth, *θ* represents the diffraction angle, *λ* is the wavelength of the X-ray source, and *K* is a dimensionless constant. The results showed a crystalline size of 38.5, 27.3, and 14.6 nm for the 1%PB-ZnO, 2%PB-ZnO, and 4%PB-ZnO samples, respectively [48]. These results agree with what was reported in TEM and are attributed to the fact that the higher the concentration of the extract used (greater quantity of organic molecules), the greater the reduction in Zinc nitrate in biosynthesis, which decreases the size of the NPs [49].

### 3.7. XPS

**Figure 7:**
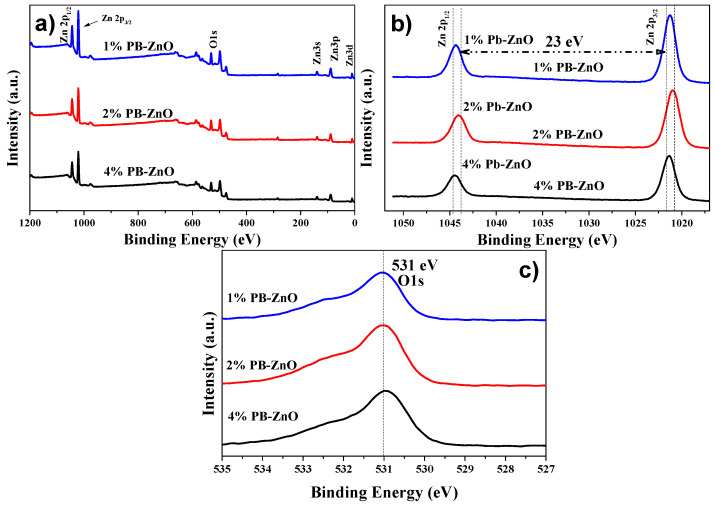
XPS of the biosynthesized ZnO NPs: (**a**) General; (**b**) Zn 2p; and (**c**) O 1s.

To complement the characterization of the ZnO NPs biosynthesized by *Peumus boldus*, XPS was used in order to perform a qualitative elemental analysis, as shown in Figure 7a, which shows the general spectra of 1%PB-ZnO, 2%PB-ZnO, and 4%PB-ZnO. The main peaks of Zn (2p1/2 at 1044.5 eV, and 2p3/2 a 1021.5 eV) and O (1s at 531 eV) were found within the three study samples. Finding these peaks confirms the successful synthesis of ZnO NPs, since, as has been reported in previous works, these peaks are characteristic of this nanomaterial [50]. Moreover, to better understand the chemical environment of the NPs, high-resolution analysis was performed for the Zn 2p and O 1s peaks. In the case of the Zn 2p peak shown in Figure 7b, a splitting of the peak into two signals was found, one at 1044.5 eV, and another at 1021.5 eV, which are assigned to the Zn 2p1/2 and Zn 2p3/2 electrons, respectively, with an energy difference of 23 eV. These peaks and the difference between them confirm the existence of Zn with an oxidation state of ^2+^, the oxidation state belonging to ZnO NPs [51]. The analysis of O 1s is observed in Figure 7c, where for the three samples there is a single peak located at 531 eV, which is due to the O1s electrons bound within the Wurtzite phase of ZnO [52].

**Figure 8:**
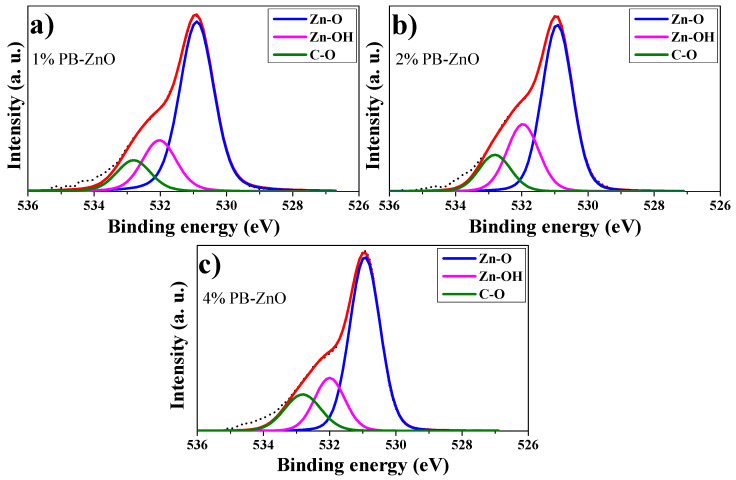
O 1s peak deconvolutions: (**a**) 1%PB-ZnO; (**b**) 2%PB-ZnO; and (**c**) 4%PB-ZnO.

Extending the analysis for a better understanding, the deconvolution of the O 1s peaks of 1%PB-ZnO, 2%PB-ZnO, and 4%PB-ZnO was performed, which is shown in Figure 8a–c, respectively. For the deconvolutions, three signals were found: One at 531 eV which is assigned to the Zn-O bond, which belongs to the ZnO nanoparticles indicating that they were obtained satisfactorily; another signal at 532 eV that is due to the Zn-OH bond related to the oxygen vacancies of the wurtzite phase of the ZnO nanoparticles; a third signal at 533 belongs to the C-O bond [53]. This link is present in the organic molecules of the *Peumus boldus* extracts, which indicates that these molecules of the natural extract are found in the NPs (verified with ATR-IR and TGA analysis). The XPS corroborated the synthesis of the ZnO NPs in the 1%PB-ZnO, 2%PB-ZnO, and 4%PB-ZnO samples, and that these also contain organic molecules from the *Peumus boldus* extracts.

### 3.8. Band Gap

Extending the characterization of the 1%PB-ZnO, 2%PB-ZnO, and 4%PB-ZnO NPs, the band gap was obtained from the UV-Vis absorbance spectrum. The TAUC model was implemented, which shows that the optical absorption strength of a nanoparticle depends on the variance between the energy of the photon and the band gap by means of the formula (Equation (2)):

(2) $${\left(\alpha hv\right)}^{1/n}=A(hv-E_{g})$$

**Figure 9:**
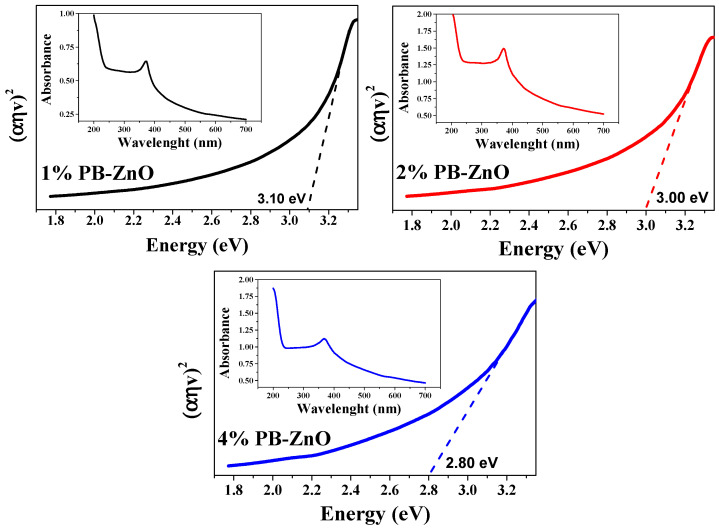
Band Gap and absorbances of the ZnO NPs biosynthesized with *Peumus boldus*.

In the previous model, *α* is the absorption coefficient, *h* is the value of Planck’s constant, *n* is the frequency of the photon, *Eg* is the band gap, and *A* is a constant of proportionality. The value of *n* is defined by the nature of the electronic transition; whether it is permitted or prohibited, direct or indirect [54], the results are shown in Figure 9. The band gap values found were 3.10, 3.00, and 2.80 eV for 1%PB-ZnO, 2%PB-ZnO, and 4%PB-ZnO, respectively. These values are very similar to those of conventional NPs [55], but there is a noticeable downward shift as the amount of *Peumus boldus* used increases. This effect has already been reported previously [56]. According to previous reports, this is due to the incorporation of organic materials in the NPs. This incorporation causes a photosensitization effect to be carried out, which consists of adding a photosensitizing material (organic molecules from *Peumus boldus*) to the NPs, making them able to be excited with a longer wavelength (lower energy) [57,58]. The reduction in the band gap is beneficial for the application of NPs in the photocatalytic degradation of pollutants.

### 3.9. ZnO NPs Formation Mechanism

**Figure 10:**
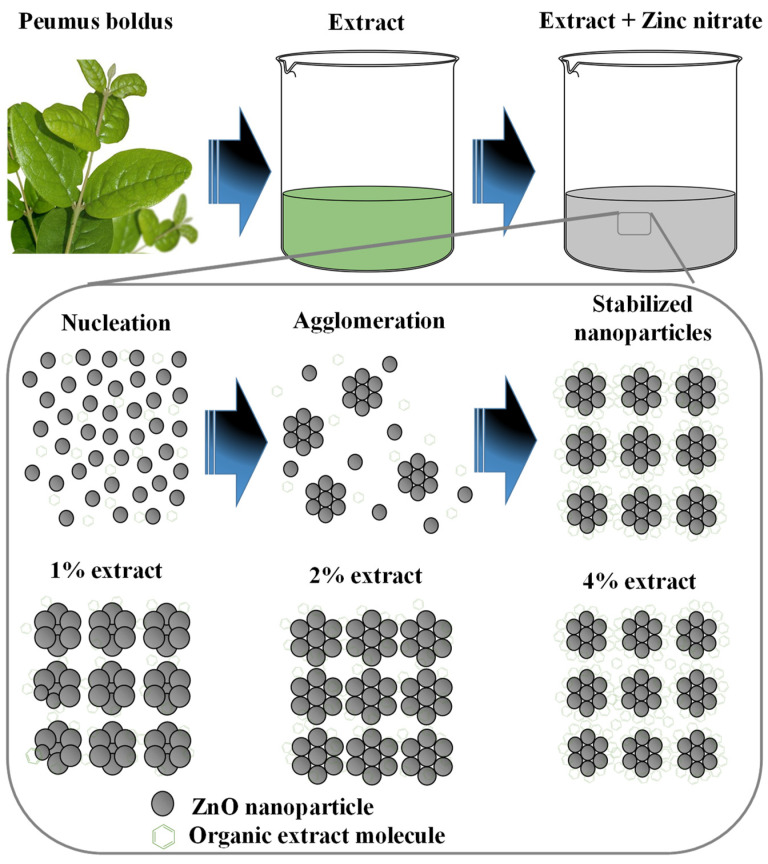
Mechanism of formation of ZnO NPs through green synthesis.

Figure 10 shows the formation process of ZnO NPs through green synthesis. The first step is to obtain the *Peumus boldus* extract (process presented in the methodology section), then the metal salt (Zn(NO_3_)_2_·6H_2_O) is incorporated into the extract, which initiates the hydrolysis reaction when stirred with the water molecules present in the extract [59]. Due to hydrolysis, the nucleation process of what will be the NPs begins, followed by the agglomeration of atoms on the generated nuclei, forming the NPs. In this process, the organic molecules of the extract play a very important role, since they can bind through OH interactions to the surface of the ZnO NPs, serving as stabilizing agents, and avoiding over-agglomeration and excessive growth of the NPs [60,61]. This is why, by adding a higher percentage of extract in the biosynthesis of NPs, the sizes decrease, and less agglomeration is observed (SEM and TEM studies). Lastly, there is the stabilization process. In the end, the ZnO NPs go through a heat treatment at 400 °C, which causes them to crystallize, but the functionalized molecules of the extracts in the NPs provide greater thermal stability (TGA Analysis) [62]. This is why it is possible to appreciate in the ATR-IR study some molecular vibrations originating from the *Peumus boldus* extract.

### 3.10. Photocatalytic Activity

#### 3.10.1. MB Degradation

**Figure 11:**
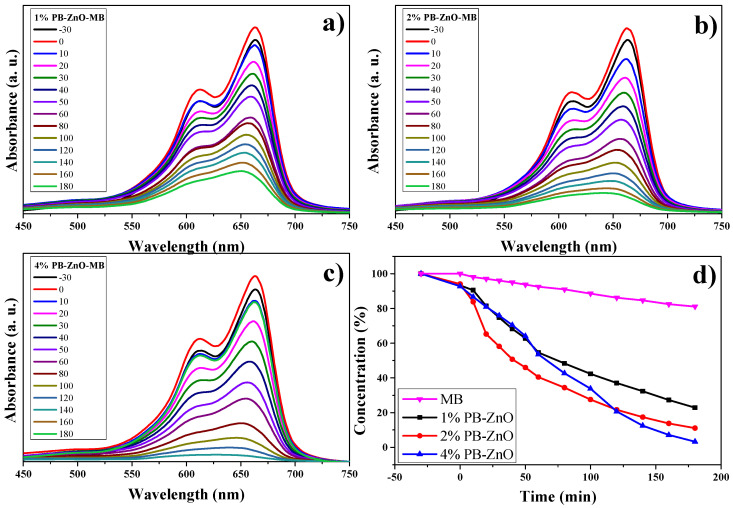
Photocatalytic study of ZnO NPs in the removal of MB.

The study of the photocatalytic degradation of the MB dye for three hours under UV radiation is shown in Figure 11. The absorption results, using UV-Vis equipment, show the maximum concentration peak at 665 nm [63] as time goes by. Figure 11a–c show the degradation of MB using the 1%PB-ZnO, 2%Bb-ZnO, and 4%PB-ZnO samples, respectively. The concentration of MB decreases according to the amount of extract that was used to synthesize the ZnO NPs, showing that the 4%PB-ZnO sample achieved total degradation (concentration equal to = 0%). The summary of these results can be seen in Figure 11d, where a reaction of the dye can also be seen without the presence of ZnO NPs, which showed a very slow natural degradation; after three hours, only 19% of the dye was degraded. There are previous studies that have shown that MB degrades over a long time. The study by Adeleke et al. in 2018 showed degradation of only approximately 6% of the MB in 70 min without the presence of any catalyst [64]. Similarly, Anju Chanu et al. presented an MB degradation of 2% in 120 min without the presence of a catalyst in 2019 [65].

Using ZnO NPs as catalysts in the dye degradation process is a very good alternative since 78% and 89% of the MB were degraded within three hours with the 1%PB-ZnO and 2%PB-ZnO samples, respectively. Moreover, the most outstanding sample was that of 4%PB-ZnO since it managed to degrade the contaminating MB completely in 180 min. These results demonstrate the effectiveness of the semiconductor NPs biosynthesized with *Peumus boldus* as catalysts in the removal of organic dyes.

#### 3.10.2. MO Degradation

**Figure 12:**
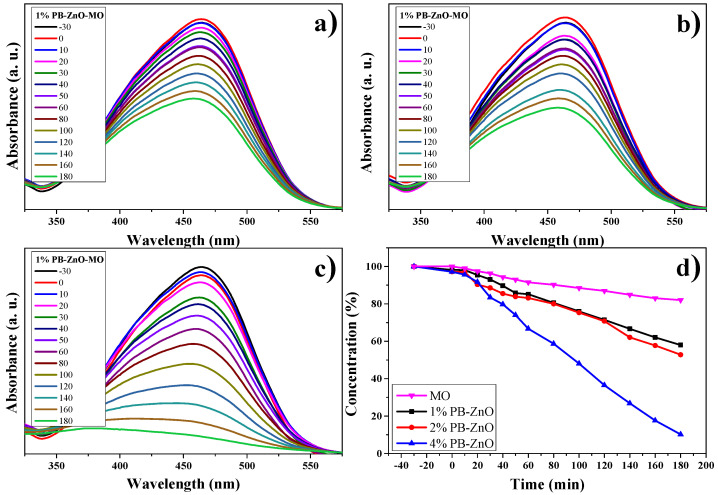
Photocatalytic study of ZnO NPs in MO removal.

The results of the OM degradation study are shown in Figure 12. The highest absorbance in the UV-Vis spectrum of the organic dye is approximately between 350 and 550 nm; therefore, this absorption band was taken as a reference for the analysis. Figure 12a–c present said band and the evolution of the photocatalytic degradation that they had during the study. As the exposure time to UV radiation passed, the concentration of the dye decreased, but the 4%PB-ZnO sample is the one that showed the highest photocatalytic activity in the degradation of the contaminant. The summary of the data is observed in Figure 12d, where the percentage of degradation of the solutions according to time is presented. It should be noted that the removal of MO without the presence of a catalyst was low, managing to degrade only 18% in three hours under solar radiation. This behavior is similar to other previously reported studies [66]. The degradation of MO under UV radiation has also been reported at only 6.4% after six hours [67].

As seen in Figure 12d, a clear trend is presented: The greater the amount of extract used in the biosynthesis of ZnO NPs, the greater its photocatalytic activity. The degradations, after 3 h, were 42% and 47% for the 1%PB-ZnO and 2%PB-ZnO samples, respectively, while the 4%PB-ZnO sample managed to degrade 92% of the dye within 180 min, which is attributed to the fact that the organic molecules present in the NPs help to reduce their band gap (Figure 9), since they act as photosensitizers, causing an electronically excited state and helping to produce electronic transition in the system. This leads to greater photocatalytic degradation of the dye (See degradation mechanism).

#### 3.10.3. RhB Degradation

**Figure 13:**
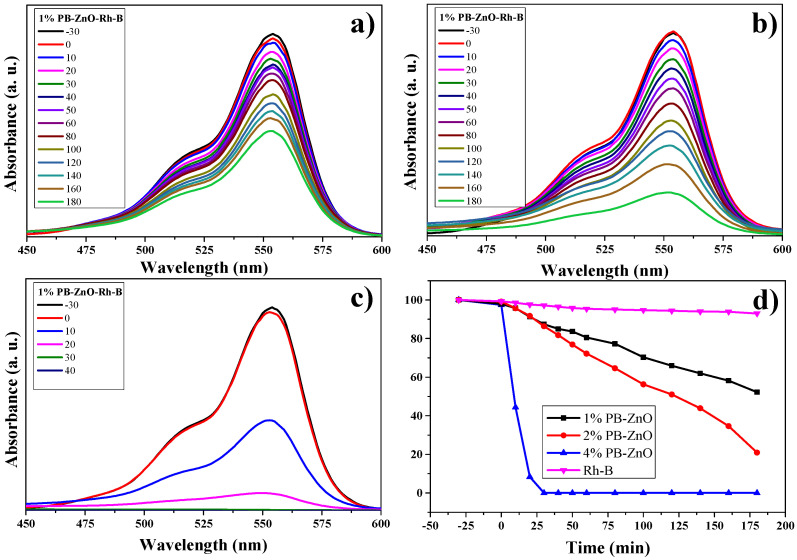
Photocatalytic study of ZnO NPs in the degradation of RhB.

The RhB degradation study is observed in Figure 13. The absorbance spectra of the three different concentrations of ZnO NPs are observed in Figure 13a–c. The maximum absorption intensity of the dye occurs between approximately 500 and 575 nm [68,69]. As the percentage of *Peumus boldus* used in the biosynthesis of NPs increases. This is attributed to the organic molecules acting as photosensitizers [70]. The results showed that the dye without a catalyst degraded only 8% in three hours; other studies have shown a 6% degradation of RhB without the presence of a catalyst in 150 min [71]. On the other hand, the 1%PB-ZnO sample managed to degrade 49% in three hours, while the 2%PB-ZnO sample degraded 80%. Finally, the 4%PB-ZnO sample degraded 100% of the contaminant in 30 min. The experiments were carried out twice, obtaining the same result (Figure 13d). It should be noted that, for the 4%PB-ZnO sample, the photocatalytic degradation of the dye is greater due to the number of organic molecules present in the NPs in addition to the anionic nature of the dye, which causes greater electronic movement in the system and, therefore, better degradation. Table 2 presents the results of other studies on the green synthesis of ZnO NPs, and their photocatalytic activity in the removal of MB, MO, and RhB.

### 3.11. Photocatalytic Mechanism

**Figure 14:**
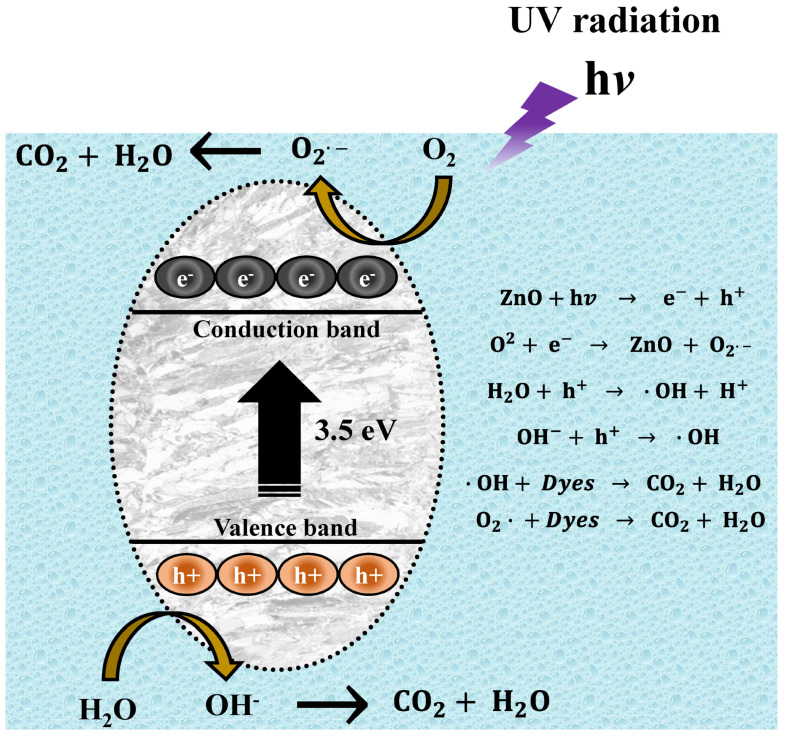
Mechanism of photocatalytic degradation of organic dyes.

For the present experiments, a proposal for a photodegradation mechanism is made, which is shown in Figure 14. In order for the contaminant degradation process to start, first the ZnO NPs have to be in a contaminated aqueous medium (MB, MO, and RhB), then the solution has to be irradiated with UV energy. After light irradiation with sufficient energy, the NPs’ energy gap (band gap) is overcome by the photoexcited electrons, which move from the valence band (VB) to the conduction band (CB). This causes electron-hole pairs to form. These electron holes react with water (H_2_O) in the aqueous medium, forming protons (H^+^) and hydroxyl radicals (OH^−^). On the other hand, the conduction band electrons react with the O_2_ present in the water, generating superoxide radicals (O_2_^−^). The radicals generated in this process are highly oxidative and seek their stability by attacking the dye molecule. This process occurs repeatedly, as long as the UV light continues its incidence until the organic dye molecule is broken down into CO_2_ y H_2_O [80].

## 4. Conclusions

This research confirms that the synthesis of ZnO NPs is possible with the help of the natural extract of *Peumus boldus*. The NPs obtained presented quasispherical morphologies with average sizes of 14 to 30 nm. ATR-IR and TGA analysis showed that the organic molecules of the natural extract remained on the surface of the NPs, this presence causes modifications in the properties of the NPs, which are beneficial for numerous applications since they help in certain processes. An important modification of the properties is the band gap value which reached values from 2.8 to 3.1 eV. These values suggest that they can be applied in photocatalysis as demonstrated in this report; the values are lower than those reported in the literature for commercial ZnO, suggesting that they could be used in photocatalysis under solar radiation in future applications. The results of the photocatalytic tests show that 4% PB-ZnO (made with 4% extract) degraded 100% of MB and MO in 180 min, and 100% of RhB in 30 min. Therefore, it was verified that the ZnO NPs biosynthesized with *Peumus boldus* can function as catalysts in the photocatalytic process. Therefore, this research serves as the basis for the generation of water treatment plants or to take this experiment to an industrial scale.

## Figures and Tables

**Table 1:** BET Surface area, pore size, and volume of the ZnO NPs.

Sample	Surface Area (m^2^/g)	Pore Volume (Å)	Pore Size (Å)
1% PB-ZnO	1.813	0.002	37.65
2% PB-ZnO	4.644	0.005	29.10
4% PB-ZnO	8.348	0.018	15.76

**Table 2:** Studies on the photocatalytic activity of ZnO nanoparticles in the removal of organic dyes.

Year	Reducing Agent	Nanoparticles	Organic Pollutant	Photocatalytic Activity	Reference
2022	*Peumus boldus*	ZnO	MB	96% in 180 min.	[72]
2021	*Syzygium cumini*	ZnO	MB	91.4% in 180 min.	[73]
2019	*Scutellaria baicalensis*	ZnO	MB	98.6% in 210 min.	[74]
2021	*Capsicum annuum var. Anaheim*	ZnO	MB	100% in 60 min.	[28]
2023	***Peumus boldus***	ZnO	MB	100% in 180 min.	Current Study
2021	*Chlorophytum comosum*	ZnO	MO	77% in 6 h.	[75]
2021	*Hedyotis capitellata*	Ce-doped ZnO	MO	92% in 240 min.	[76]
2022	*Hagenia abyssinica*	ZnO	MO	83.17% in 120 min.	[77]
2021	*Capsicum annuum var. Anaheim*	ZnO	MO	85% in 180 min.	[28]
2023	***Peumus boldus***	ZnO	MO	92% in 180 min.	Current Study
2022	*Sechium edule polysaccharides*	ZnO	RhB	95% in 75 min.	[78]
2022	*Sapindus rarak*	ZnO	RhB	99.7% in 120 min.	[79]
2019	*Cyanometra ramiflora*	ZnO	RhB	98% in 200 min.	[45]
2021	*Capsicum annuum var. Anaheim*	ZnO	RhB	92% in 180 min.	[28]
2023	***Peumus boldus***	ZnO	RhB	100% in 30 min.	Current Study

## Data Availability

The most important data from this research are available in this article.

## References

[1] Shahrajabian M.H., Sun W., Cheng Q. (2019). The power of natural Chinese medicine, ginger and ginseng root in an organic life. Middle-East J. Sci. Res..

[2] Oszmiański J., Wojdyło A., Juszczyk P., Nowicka P. (2020). Roots and Leaf Extracts of *Dipsacus fullonum* L. and Their Biological Activities. Plants.

[3] Idris O.A., Wintola O.A., Afolayan A.J. (2019). Evaluation of the Bioactivities of *Rumex crispus* L. Leaves and Root Extracts Using Toxicity, Antimicrobial, and Antiparasitic Assays. Evid.-Based Complement. Altern. Med..

[4] Blanco Y.D., Campos E.C.M., Valdés C.I.R., Chavarín J.U. (2019). Natural additive (nopal mucilage) on the electrochemical properties of concrete reinforcing steel. Rev. ALCONPAT.

[5] Chávez J.R.B., Martínez F.C., Soto J.R., Sierra A.D., Celaya M.V. (2019). Comportamiento de mezclas de mortero con residuos de mármol (polvo), cáscara de nuez y mucílago de nopal. Rev. Arquit. E Ing..

[6] Shariza M.A., Philip D.C., Maszura S.M.S. (2019). Preliminary Study on Properties of Oil Palm Shell Lightweight Concrete with Cockle Shell as Mixing Ingredient. IOP Conf. Ser. Mater. Sci. Eng..

[7] Kouhbanani M.A.J., Beheshtkhoo N., Fotoohiardakani G., Hosseini-Nave H., Taghizadeh S., Amani A.M. (2019). Green Synthesis and Characterization of Spherical Structure Silver Nanoparticles Using Wheatgrass Extract. J. Environ. Treat. Tech..

[8] Pacheco-Fernández I., Pino V. (2019). Green solvents in analytical chemistry. Curr. Opin. Green Sustain. Chem..

[9] Rafique M., Sadaf I., Rafique M.S., Tahir M.B. (2017). A review on green synthesis of silver nanoparticles and their applications. Artif. Cells Nanomed. Biotechnol..

[10] Mir B.A., Rasool S., Rehman M.U., Amin I., Ali R. (2019). Anticancer Mechanistic Insights of Epigallocatechin-3-Gallate, an Active Ingredient of Green Tea (*Camellia sinensis*). Plant and Human Health.

[11] Choe M.-S., Jung C.-H., Park J.-H., Yoon W.-K., Min B.-T., Yoo M. (2019). Polyphenol, Flavonoid and Tannin Variation in Puer Tea Extracts. Quant. Bio-Sci..

[12] Yadi M., Mostafavi E., Saleh B., Davaran S., Aliyeva I., Khalilov R., Nikzamir M., Nikzamir N., Akbarzadeh A., Panahi Y. (2018). Current developments in green synthesis of metallic nanoparticles using plant extracts: A review. Artif. Cells Nanomed. Biotechnol..

[13] Nabi G., Raza W., Tahir M.B. (2019). Green Synthesis of TiO_2_ Nanoparticle Using Cinnamon Powder Extract and the Study of Optical Properties. J. Inorg. Organomet. Polym. Mater..

[14] Luque P.A., Nava O., Soto-Robles C.A., Garrafa-Galvez H.E., Martínez-Rosas M.E., Chinchillas-Chinchillas M.J., Vilchis-Nestor A.R., Castro-Beltrán A. (2020). SnO_2_ nanoparticles synthesized with Citrus aurantifolia and their performance in photocatalysis. J. Mater. Sci. Mater. Electron..

[15] Fuentes-Barros G., Castro-Saavedra S., Liberona L., Acevedo-Fuentes W., Tirapegui C., Mattar C., Cassels B.K. (2018). Variation of the alkaloid content of Peumus boldus (boldo). Fitoterapia.

[16] Girardi N.S., Passone M.A., García D., Nesci A., Etcheverry M. (2018). Microencapsulation of Peumus boldus essential oil and its impact on peanut seed quality preservation. Ind. Crops Prod..

[17] Otero C., Miranda-Rojas S., Llancalahuén F.M., Fuentes J.A., Atala C., González-Silva G., Verdugo D., Sierra-Rosales P., Moreno A., Gordillo-Fuenzalida F. (2022). Biochemical characterization of Peumus boldus fruits: Insights of its antioxidant properties through a theoretical approach. Food Chem..

[18] Dey A. (2018). Semiconductor metal oxide gas sensors: A review. Mater. Sci. Eng. B.

[19] Charbgoo F., Ahmad M.B., Darroudi M. (2017). Cerium oxide nanoparticles: Green synthesis and biological applications. Int. J. Nanomed..

[20] Dhand V., Soumya L., Bharadwaj S., Chakra S., Bhatt D., Sreedhar B. (2016). Green synthesis of silver nanoparticles using Coffea arabica seed extract and its antibacterial activity. Mater. Sci. Eng. C.

[21] Subhapriya S., Gomathipriya P. (2018). Green synthesis of titanium dioxide (TiO_2_) nanoparticles by Trigonella foenum-graecum extract and its antimicrobial properties. Microb. Pathog..

[22] Arshad M., Ehtisham-ul-Haque S., Bilal M., Ahmad N., Ahmad A., Abbas M., Nisar J., Khan M.I., Nazir A., Ghaffar A. (2020). Synthesis and characterization of Zn doped WO_3_ nanoparticles: Photocatalytic, antifungal and antibacterial activities evaluation. Mater. Res. Express.

[23] Şahan H., Göktepe H., Yıldız S., Çaymaz C., Patat Ş. (2019). A novel and green synthesis of mixed phase CoO@Co_3_O_4_@C anode material for lithium ion batteries. Ionics.

[24] Luque P.A., Nava O., Soto-Robles C.A., Chinchillas-Chinchillas M.J., Garrafa-Galvez H.E., Baez-Lopez Y.A., Valdez-Núñez K.P., Vilchis-Nestor A.R., Castro-Beltrán A. (2020). Improved photocatalytic efficiency of SnO_2_ nanoparticles through green synthesis. Optik.

[25] Luque P.A., Soto-Robles C.A., Nava O., Gomez-Gutierrez C.M., Castro-Beltran A., Garrafa-Galvez H.E., Vilchis-Nestor A.R., Olivas A. (2018). Green synthesis of zinc oxide nanoparticles using Citrus sinensis extract. J. Mater. Sci. Mater. Electron..

[26] Ahmadi Shadmehri A., Namvar F. (2020). A Review on Green Synthesis, Cytotoxicity Mechanism and Antibacterial Activity of Zno-NPs. Int. J. Res. Appl. Basic Med. Sci..

[27] Tabrizi Hafez Moghaddas S.M., Elahi B., Javanbakht V. (2020). Biosynthesis of pure zinc oxide nanoparticles using Quince seed mucilage for photocatalytic dye degradation. J. Alloys Compd..

[28] Luque-Morales P.A., Lopez-Peraza A., Nava-Olivas O.J., Amaya-Parra G., Baez-Lopez Y.A., Orozco-Carmona V.M., Garrafa-Galvez H.E., Chinchillas-Chinchillas M.d.J. (2021). ZnO Semiconductor Nanoparticles and Their Application in Photocatalytic Degradation of Various Organic Dyes. Materials.

[29] Keshipour S., Mohammad-Alizadeh S., Razeghi M.H. (2022). Copper phthalocyanine@graphene oxide as a cocatalyst of TiO_2_ in hydrogen generation. J. Phys. Chem. Solids.

[30] Zhang P., Lou X.W. (2019). Design of Heterostructured Hollow Photocatalysts for Solar-to-Chemical Energy Conversion. Adv. Mater..

[31] Wang Y., Yang H., Sun X., Zhang H., Xian T. (2020). Preparation and photocatalytic application of ternary n-BaTiO_3_/Ag/p-AgBr heterostructured photocatalysts for dye degradation. Mater. Res. Bull..

[32] Mohanraj J., Durgalakshmi D., Balakumar S., Aruna P., Ganesan S., Rajendran S., Naushad M. (2020). Low cost and quick time absorption of organic dye pollutants under ambient condition using partially exfoliated graphite. J. Water Process Eng..

[33] Hasanzadeh M., Simchi A., Far H.S. (2020). Nanoporous composites of activated carbon-metal organic frameworks for organic dye adsorption: Synthesis, adsorption mechanism and kinetics studies. J. Ind. Eng. Chem..

[34] Ismail A.M., Menazea A.A., Kabary H.A., El-Sherbiny A.E., Samy A. (2019). The influence of calcination temperature on structural and antimicrobial characteristics of zinc oxide nanoparticles synthesized by Sol–Gel method. J. Mol. Struct..

[35] Shamsuzzaman A.A., Asif M., Mashrai A., Khanam H. (2014). Green Synthesis of ZnO Nanoparticles Using Bacillus Subilis And Their Catalytic Performance In The One-Pot Synthesis of Steroidal Thiophenes. Eur. Chem. Bull..

[36] Umavathi S., Mahboob S., Govindarajan M., Al-Ghanim K.A., Ahmed Z., Virik P., Al-Mulhm N., Subash M., Gopinath K., Kavitha C. (2021). Green synthesis of ZnO nanoparticles for antimicrobial and vegetative growth applications: A novel approach for advancing efficient high quality health care to human wellbeing. Saudi J. Biol. Sci..

[37] Al-Ajmi M.F., Hussain A., Alsharaeh E., Ahmed F., Amir S., Anwar M.S., Siddiqui M.A., Al-Khedhairy A.A., Koo B.H. (2018). Green synthesis of zinc oxide nanoparticles using alstonia macrophylla leaf extract and their in-vitro anticancer activity. Sci. Adv. Mater..

[38] Matinise N., Fuku X.G., Kaviyarasu K., Mayedwa N., Maaza M. (2017). ZnO nanoparticles via Moringa oleifera green synthesis: Physical properties & mechanism of formation. Appl. Surf. Sci..

[39] Golmohammadi M., Honarmand M., Ghanbari S. (2020). A green approach to synthesis of ZnO nanoparticles using jujube fruit extract and their application in photocatalytic degradation of organic dyes. Spectrochim. Acta Part A Mol. Biomol. Spectrosc..

[40] Luque P.A., Garrafa-Gálvez H.E., Nava O., Olivas A., Martínez-Rosas M.E., Vilchis-Nestor A.R., Villegas-Fuentes A., Chinchillas-Chinchillas M.J. (2021). Efficient sunlight and UV photocatalytic degradation of Methyl Orange, Methylene Blue and Rhodamine B, using Citrus×paradisi synthesized SnO2 semiconductor nanoparticles. Ceram. Int..

[41] Yadav L.S.R., Raghavendra M., Manjunath K., Nagaraju G. (2018). Photocatalytic, biodiesel, electrochemical sensing properties and formylation reactions of ZnO nanoparticles synthesized via eco-friendly green synthesis method. J. Mater. Sci. Mater. Electron..

[42] Singh K., Singh J., Rawat M. (2019). Green synthesis of zinc oxide nanoparticles using Punica Granatum leaf extract and its application towards photocatalytic degradation of Coomassie brilliant blue R-250 dye. SN Appl. Sci..

[43] Goutam S.P., Saxena G., Singh V., Yadav A.K., Bharagava R.N., Thapa K.B. (2018). Green synthesis of TiO_2_ nanoparticles using leaf extract of *Jatropha curcas* L. for photocatalytic degradation of tannery wastewater. Chem. Eng. J..

[44] Mohanta D., Ahmaruzzaman M. (2016). Tin oxide nanostructured materials: An overview of recent developments in synthesis, modifications and potential applications. RSC Adv..

[45] Varadavenkatesan T., Lyubchik E., Pai S., Pugazhendhi A., Vinayagam R., Selvaraj R. (2019). Photocatalytic degradation of Rhodamine B by zinc oxide nanoparticles synthesized using the leaf extract of Cyanometra ramiflora. J. Photochem. Photobiol. B Biol..

[46] López-López J., Tejeda-Ochoa A., López-Beltrán A., Herrera-Ramírez J., Méndez-Herrera P. (2022). Sunlight Photocatalytic Performance of ZnO Nanoparticles Synthesized by Green Chemistry Using Different Botanical Extracts and Zinc Acetate as a Precursor. Molecules.

[47] El-Belely E.F., Farag M.M.S., Said H.A., Amin A.S., Azab E., Gobouri A.A., Fouda A. (2021). Green Synthesis of Zinc Oxide Nanoparticles (ZnO-NPs) Using Arthrospira platensis (Class: Cyanophyceae) and Evaluation of their Biomedical Activities. Nanomaterials.

[48] Ishwarya R., Vaseeharan B., Kalyani S., Banumathi B., Govindarajan M., Alharbi N.S., Kadaikunnan S., Al-anbr M.N., Khaled J.M., Benelli G. (2018). Facile green synthesis of zinc oxide nanoparticles using Ulva lactuca seaweed extract and evaluation of their photocatalytic, antibiofilm and insecticidal activity. J. Photochem. Photobiol. B Biol..

[49] Chikkanna M.M., Neelagund S.E., Rajashekarappa K.K. (2018). Green synthesis of Zinc oxide nanoparticles (ZnO NPs) and their biological activity. SN Appl. Sci..

[50] Sun Y., Zong Y., Feng J., Li X., Yan F., Lan Y., Zhang L., Ren Z., Zheng X. (2018). Oxygen vacancies driven size-dependent d0 room temperature ferromagnetism in well-dispersed dopant-free ZnO nanoparticles and density functional theory calculation. J. Alloys Compd..

[51] Khan M.M., Saadah N.H., Khan M.E., Harunsani M.H., Tan A.L., Cho M.H. (2019). Potentials of Costus woodsonii leaf extract in producing narrow band gap ZnO nanoparticles. Mater. Sci. Semicond. Process..

[52] Liu Y., Zhang Q., Xu M., Yuan H., Chen Y., Zhang J., Luo K., Zhang J., You B. (2019). Novel and efficient synthesis of Ag-ZnO nanoparticles for the sunlight-induced photocatalytic degradation. Appl. Surf. Sci..

[53] Gandla S., Gollu S.R., Sharma R., Sarangi V., Gupta D. (2015). Dual role of boron in improving electrical performance and device stability of low temperature solution processed ZnO thin film transistors. Appl. Phys. Lett..

[54] Viezbicke B.D., Patel S., Davis B.E., Birnie III D.P. (2015). Evaluation of the Tauc method for optical absorption edge determination: ZnO thin films as a model system. Phys. Status Solidi.

[55] Singh J., Kaur S., Kaur G., Basu S., Rawat M. (2019). Biogenic ZnO nanoparticles: A study of blueshift of optical band gap and photocatalytic degradation of reactive yellow 186 dye under direct sunlight. Green Process. Synth..

[56] Rafique M., Tahir R., Gillani S.S.A., Tahir M.B., Shakil M., Iqbal T., Abdellahi M.O. (2022). Plant-mediated green synthesis of zinc oxide nanoparticles from Syzygium Cumini for seed germination and wastewater purification. Int. J. Environ. Anal. Chem..

[57] Keshipour S., Mohammad-Alizadeh S. (2021). Nickel phthalocyanine@graphene oxide/TiO_2_ as an efficient degradation catalyst of formic acid toward hydrogen production. Sci. Rep..

[58] Yang C., Dong W., Cui G., Zhao Y., Shi X., Xia X., Tang B., Wang W. (2017). Highly efficient photocatalytic degradation of methylene blue by P2ABSA-modified TiO_2_ nanocomposite due to the photosensitization synergetic effect of TiO_2_ and P2ABSA. RSC Adv..

[59] Herbst M., Hofmann E., Förster S. (2019). Nucleation and Growth Kinetics of ZnO Nanoparticles Studied by in Situ Microfluidic SAXS/WAXS/UV–Vis Experiments. Langmuir.

[60] Bandeira M., Giovanela M., Roesch-Ely M., Devine D.M., da Silva Crespo J. (2020). Green synthesis of zinc oxide nanoparticles: A review of the synthesis methodology and mechanism of formation. Sustain. Chem. Pharm..

[61] Chan Y.Y., Pang Y.L., Lim S., Chong W.C. (2021). Facile green synthesis of ZnO nanoparticles using natural-based materials: Properties, mechanism, surface modification and application. J. Environ. Chem. Eng..

[62] Nava O.J., Luque P.A., Gómez-Gutiérrez C.M., Vilchis-Nestor A.R., Castro-Beltrán A., Mota-González M.L., Olivas A. (2017). Influence of Camellia sinensis extract on Zinc Oxide nanoparticle green synthesis. J. Mol. Struct..

[63] Prabakaran E., Pillay K. (2019). Synthesis of N-doped ZnO nanoparticles with cabbage morphology as a catalyst for the efficient photocatalytic degradation of methylene blue under UV and visible light. RSC Adv..

[64] Adeleke J.T., Theivasanthi T., Thiruppathi M., Swaminathan M., Akomolafe T., Alabi A.B. (2018). Photocatalytic degradation of methylene blue by ZnO/NiFe_2_O_4_ nanoparticles. Appl. Surf. Sci..

[65] Anju Chanu L., Joychandra Singh W., Jugeshwar Singh K., Nomita Devi K. (2019). Effect of operational parameters on the photocatalytic degradation of Methylene blue dye solution using manganese doped ZnO nanoparticles. Results Phys..

[66] Weldegebrieal G.K., Sibhatu A.K. (2021). Photocatalytic activity of biosynthesized α-Fe_2_O_3_ nanoparticles for the degradation of methylene blue and methyl orange dyes. Optik.

[67] Chen H., Xue C., Cui D., Liu M., Chen Y., Li Y., Zhang W. (2020). Co_3_O_4_–Ag photocatalysts for the efficient degradation of methyl orange. RSC Adv..

[68] Mancipe S., Martínez J.J., Pinzón C., Rojas H., Solis D., Gómez R. (2021). Effective photocatalytic degradation of Rhodamine B using tin semiconductors over hydrotalcite-type materials under sunlight driven. Catal. Today.

[69] Rajendran A., Alsawalha M., Alomayri T. (2021). Biogenic synthesis of husked rice-shaped iron oxide nanoparticles using coconut pulp (*Cocos nucifera* L.) extract for photocatalytic degradation of Rhodamine B dye and their in vitro antibacterial and anticancer activity. J. Saudi Chem. Soc..

[70] Luque P.A., Chinchillas-Chinchillas M.J., Nava O., Lugo-Medina E., Martínez-Rosas M.E., Carrillo-Castillo A., Vilchis-Nestor A.R., Madrigal-Muñoz L.E., Garrafa-Gálvez H.E. (2021). Green synthesis of tin dioxide nanoparticles using Camellia sinensis and its application in photocatalytic degradation of textile dyes. Optik.

[71] Liu Z., He X., Yang X., Ding H., Wang D., Ma D., Feng Q. (2021). Synthesis of mesoporous carbon nitride by molten salt-assisted silica aerogel for Rhodamine B adsorption and photocatalytic degradation. J. Mater. Sci..

[72] Villarreal R.C., Luque-Morales M., Chinchillas-Chinchillas M.J., Luque P.A. (2022). Langmuir-Hinshelwood-Hougen-Watson model for the study of photodegradation properties of zinc oxide semiconductor nanoparticles synthetized by Peumus boldus. Results Phys..

[73] Sadiq H., Sher F., Sehar S., Lima E.C., Zhang S., Iqbal H.M.N., Zafar F., Nuhanović M. (2021). Green synthesis of ZnO nanoparticles from Syzygium Cumini leaves extract with robust photocatalysis applications. J. Mol. Liq..

[74] Chen L., Batjikh I., Hurh J., Han Y., Huo Y., Ali H., Li J.F., Rupa E.J., Ahn J.C., Mathiyalagan R. (2019). Green synthesis of zinc oxide nanoparticles from root extract of Scutellaria baicalensis and its photocatalytic degradation activity using methylene blue. Optik.

[75] Shaker Ardakani L., Alimardani V., Tamaddon A.M., Amani A.M., Taghizadeh S. (2021). Green synthesis of iron-based nanoparticles using Chlorophytum comosum leaf extract: Methyl orange dye degradation and antimicrobial properties. Heliyon.

[76] Nguyen-Hong Y., Luu T.V.H., Doan V. (2021). Green synthesis of Ce-doped ZnO nanoparticles using Hedyotis capitellata Leaf extract for efficient photocatalytic degradation of Methyl Orange. Vietnam J. Chem..

[77] Zewde D., Geremew B. (2022). Biosynthesis of ZnO nanoparticles using Hagenia abyssinica leaf extracts; their photocatalytic and antibacterial activities. Environ. Pollut. Bioavailab..

[78] Bharathi D., AlSalhi M.S., Devanesan S., Nandagopal J.G.T., Kim W., Ranjithkumar R. (2022). Photocatalytic degradation of Rhodamine B using green-synthesized ZnO nanoparticles from Sechium edule polysaccharides. Appl. Nanosci..

[79] Umar A., Sabrina V., Yulizar Y. (2022). Synthesis of ZnO nanoparticles using Sapindus rarak DC fruit pericarp extract for rhodamine B photodegradation. Inorg. Chem. Commun..

[80] Karthik K.V., Raghu A.V., Reddy K.R., Ravishankar R., Sangeeta M., Shetti N.P., Reddy C.V. (2022). Green synthesis of Cu-doped ZnO nanoparticles and its application for the photocatalytic degradation of hazardous organic pollutants. Chemosphere.

